# *Curcuma mangga*-Mediated Synthesis of Gold Nanoparticles: Characterization, Stability, Cytotoxicity, and Blood Compatibility

**DOI:** 10.3390/nano7060123

**Published:** 2017-05-27

**Authors:** Yiing Yee Foo, Vengadesh Periasamy, Lik Voon Kiew, G. Gnana Kumar, Sri Nurestri Abd Malek

**Affiliations:** 1 Institute of Biological Sciences, Faculty of Science, University of Malaya, Kuala Lumpur 50603, Malaysia; srimalek@yahoo.my; 2 Low Dimensional Materials Research Centre (LDMRC), Department of Physics, Faculty of Science, University of Malaya, Kuala Lumpur 50603, Malaysia; 3 Department of Pharmacology, Faculty of Medicine, University of Malaya, Kuala Lumpur 50603, Malaysia; lvkiew@um.edu.my; 4 Department of Physical Chemistry, School of Chemistry, Madurai Kamaraj University, Madurai 625021, Tamil Nadu, India; kumarg2006@gmail.com

**Keywords:** gold nanoparticles, green synthesis, in vitro stability, biocompatible, phytochemicals, hemocompatibility

## Abstract

The utilization of toxic chemicals as reducing and stabilizing agents in the preparation of gold nanoparticles (AuNPs) has increased in vivo toxicity and thus limited its application in clinical settings. Herein, we propose an alternative method of preparing highly stable AuNPs, where non-toxic *Curcuma mangga* (CM) extract was used as a single reducing and stabilizing agent to overcome the aforementioned constraints. The morphological images enunciated that the homogeneously dispersed AuNPs exhibited spherical morphology with an average particle diameter of 15.6 nm. Fourier Transform infrared (FTIR) and cyclic voltammetry analysis demonstrated that carbonyl groups of terpenoids in CM extract played an important role in the formation and stabilization of AuNPs. Green-synthesized AuNPs were found to have good stability in physiological media after 24 h of dispersion. The AuNPs were also cytocompatible with human colon fibroblast cell (CCD-18Co) and human lung fibroblast cell (MRC-5). Hemocompatibility tests revealed that the AuNPs were blood-compatible, with less than 10% of hemolysis without any aggregation of erythrocytes. The current study suggests potential in employing a CM-extract-based method in the preparation of AuNPs for anticancer diagnosis and therapy.

## 1. Introduction

In recent years, the application of gold nanoparticles (AuNPs) as a nano-diagnostic, in drug delivery, and as a therapeutic agent has received extensive attention due to the remarkable physical and chemical properties of AuNPs [1]. These includes the capability to harness energy from the tissue penetrating near infra-red radiations, excellent photothermal properties, high drug loading and the ability to functionalize with targeting moieties [2]. To date, AuNP products on clinical trials, recombinant human tumor necrosis factor alpha bound PEGylated gold nanoparticles (CYT-6091) and AuroShell particles have shown positive outcomes for cancer therapy and photothermal therapy for coronary atherosclerosis, respectively [3,4].

Only two out of thousands of AuNP formulations from the literature have progressed to human clinical trials. Poor stability in physiological environments remains one of the major limitations that contribute to poor translation of nanoparticle formulations from bench to clinic [5]. Physiological environments abundant with proteins and salts can influence the stability and interaction of AuNPs with biological systems. The high ionic strength of physiological media can cause AuNPs aggregation, thus reducing cellular internalization of AuNPs [6]. For instance, AuNPs capped with common stabilizing agents such as citrate and cetyltrimethylammonium bromide (CTAB) have been found to be unstable in buffers of pH 4, 7, 8, or 10 [7]. Thus, the stabilization of AuNPs with capping agents are often required to enable any biological applications of AuNPs [8].

Apart from the stability, biocompatibility, and biosafety of AuNPs, there are two other important factors to be considered. The synthesis of AuNPs often involves the use of toxic reducing agents or surfactants. Wan et al. reported that CTAB-coated gold nanorods (CTAB-AuNPs) are toxic to both tumors and non-malignant transformed cells at nanomolar concentrations [9]. CTAB-AuNPs have shown to aggregate on the cell membrane and cause damage to the membrane. CTAB-AuNPs also increased lysosomal membrane permeation and decreased mitochondrial membrane potential, which subsequently induced cell death [10]. Freese et al. demonstrated that the higher amount of citrate on the surface of AuNPs increases the cytotoxicity and decreases the proliferation rate of human dermal microvascular endothelial cells (HDMEC) and the human cerebral microvascular endothelial cell line (hCMEC/D3) [11]. Vijayakumar and Ganesan investigated the cytotoxic effect of the stabilizing agents of AuNPs. The citrate-stabilized AuNPs (citrate-AuNPs) were found to have increased cytotoxicity compared to starch- and gum arabic-stabilized AuNPs [12]. These studies suggested that the surface chemistry of AuNPs accounts for the biosafety of AuNPs in biological applications. Therefore, the use of non-toxic stabilizing agents in the preparation of AuNPs is necessary.

Green-synthesized AuNPs have emerged in recent times as a potential solution to overcome the stability and biocompatibility problems of chemically synthesized AuNPs. Generally, AuNPs are synthesized through physical and chemical routes, which are costly, consume high amounts of energy, and produce toxic wastes [13]. In recent years, the green synthesis of AuNPs has emerged as an alternative method due to its simplicity, cost, and energy effectiveness and considerable reduction in the generation of hazardous waste [13]. Green synthesis utilizes natural biomolecules such as polysaccharides [14], microbial enzymes [15], and phytochemicals [16] as the reducing and stabilizing agents in the formation of AuNPs. In contrast to physical and chemical synthesis, this process normally does not require heat or pressure, which ultimately reduces the energy usage and therefore lowers the production cost [17]. 

In this study, we demonstrated the synthesis of AuNPs using *Curcuma mangga* aqueous ethanol (CM) extract through a simple, one-step method. *C. mangga* is a type of ginger, traditionally used in the treatment for fever, stomachaches, and cancer [18]. It has also been reported extensively for its anticancer [18], antioxidant [19], and antimicrobial properties [20]. Numerous phytochemicals with antioxidant activity have been reported to be present in *C. mangga* [19], which could act as the reducing agent for reducing precursor Au^3+^ ions. Upon AuNP synthesis, we further investigate physico-chemical characteristics, the in vitro stability in physiological conditions, cytocompatibility, and blood compatibility.

## 2. Results

### 2.1. Characterization of AuNPs by Ultraviolet-Visible (UV-Vis) Spectroscopy and Transmission Electron Microscopy

**Figure 1:**
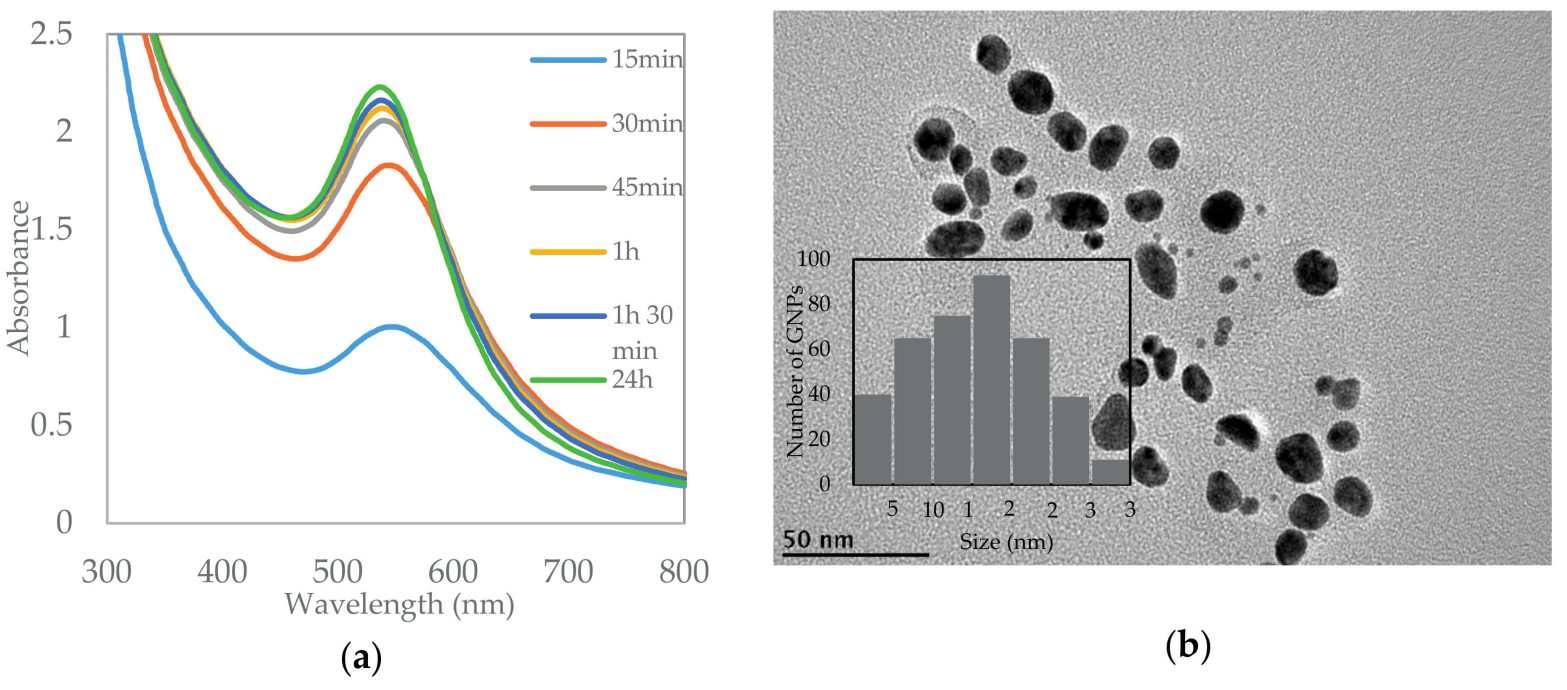
(**a**) Ultraviolet-visible (UV-Vis) spectra of gold nanoparticles (AuNPs) at incubation times of 15, 30, 45, 60, and 90 min and 24 h; (**b**) Transmission electron micrographs of AuNPs synthesized from *Curcuma mangga* (CM) extract at magnification 100,000×. (Inlet) Size distribution of AuNPs analyzed for about 400 particles using Image J.

The formation of AuNPs can be observed as the change in color from yellow to red-violet. The red-violet color observed was a result of the surface plasmon resonance (SPR) of AuNPs, which is attributed to the oscillation of free conducting electrons induced by the electromagnetic field [21]. UV-Vis spectroscopy was carried out to ascertain the formation, and to monitor the stability, of AuNPs. Figure 1a shows the UV-Vis spectra of AuNPs at different incubation times. An absorption peak at a visible wavelength of 535 nm was observed after 15 min of incubation, which corresponds to the SPR band of AuNPs. The intensity of the absorption peak increased significantly for the first hour of incubation. There was no significant increase in the intensity of the SPR band after a further 24 h of incubation, indicating the completion of a reaction at one hour of incubation. The yield of AuNPs synthesized by CM extract was found to be 52.5% using microwave plasma-atomic emission spectroscopy (MP-AES).

The size and shape of AuNPs were determined using transmission electron microscopy (TEM). TEM image (Figure 1b) illustrates that the AuNPs synthesized from CM extract were monodispersed and predominantly spherical in shape. The core sizes of AuNPs were in the range of 2–35 nm, with an average size of 15.6 nm. It was also observed that the sizes of AuNPs were distributed normally as shown in the histogram in Figure 1b (inlet). Studies have shown that nanoparticles less than 5 nm easily undergo renal clearance [22], while particles more than 200 nm will be retained in the spleen [23]. Thus, AuNPs with sizes in the range of 10–200 nm will be highly desirable for systemic administration.

### 2.2. Fourier Transform Infrared Spectroscopy 

**Figure 2:**
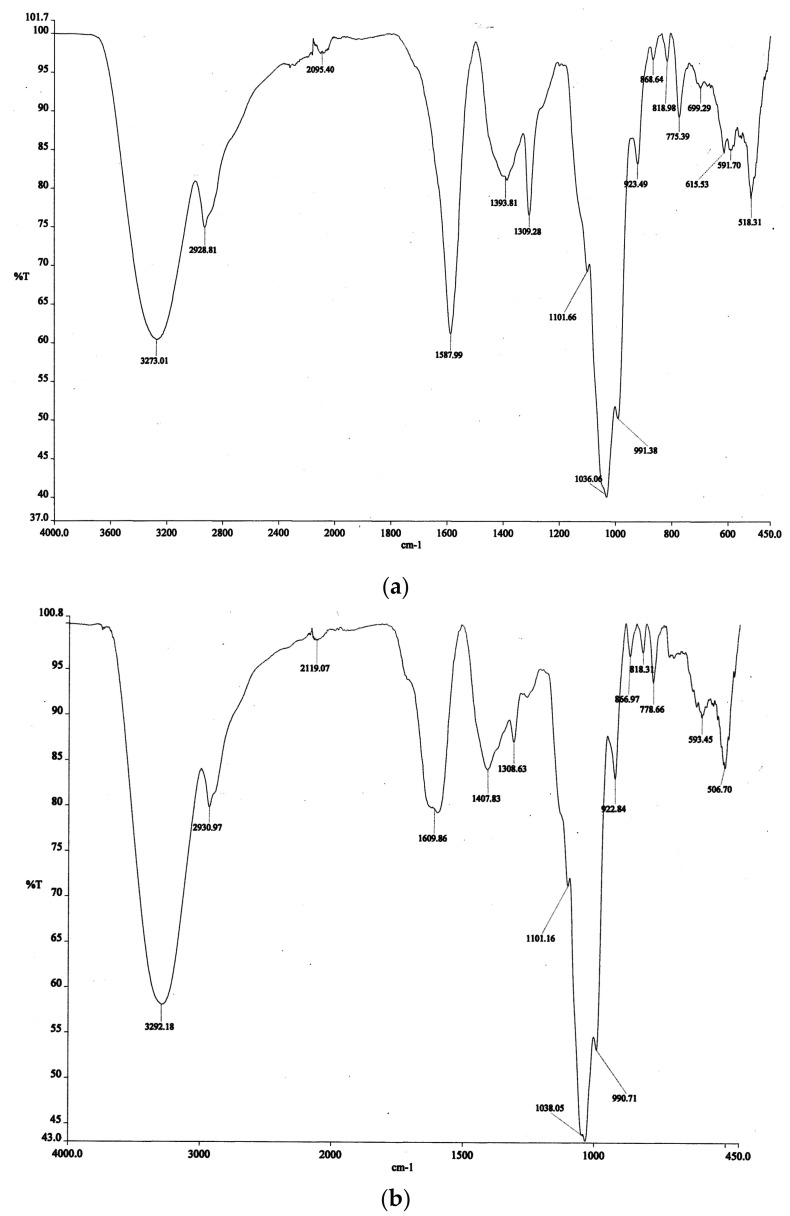
Fourier Transform infrared (FTIR) spectra of CM extract (**a**) before and (**b**) after the bioreduction of Au^3+^ ions.

Fourier Transform infrared (FTIR) spectroscopy was conducted to investigate the possible functional groups involved in the formation of AuNPs. Two intense bands were observed at 1036 and 3273 cm^−1^ in the FTIR spectra of CM before and after bioreduction of Au^3+^ (Figure 2a,b), which corresponds to C–OH and O–H bonds respectively [24]. The results support the chemical compositions of CM extract, where the C–OH and O–H bonds can be found in polyphenolics, curcuminoids, and terpenoids [18]. Other bands that are visible in both spectra (Figure 2a,b) are at 2929, 1394, and 1309 cm^−1^. Bands observed at ~500–868 cm^−1^ signify the C–H groups that are abundant in CM extract. Intense bands at 1588 cm^−1^ in the FTIR spectrum of CM extract before bioreduction was related to the unsaturated carbonyl group in terpenoids. The intensity of the band was reduced and shifted to 1610 cm^−1^ after the bioreduction of Au^3+^, suggesting that the carbonyl group of terpenoids in the CM extract reduces Au^3+^ in the synthesis of AuNPs.

### 2.3. Cyclic Voltammetry

**Figure 3:**
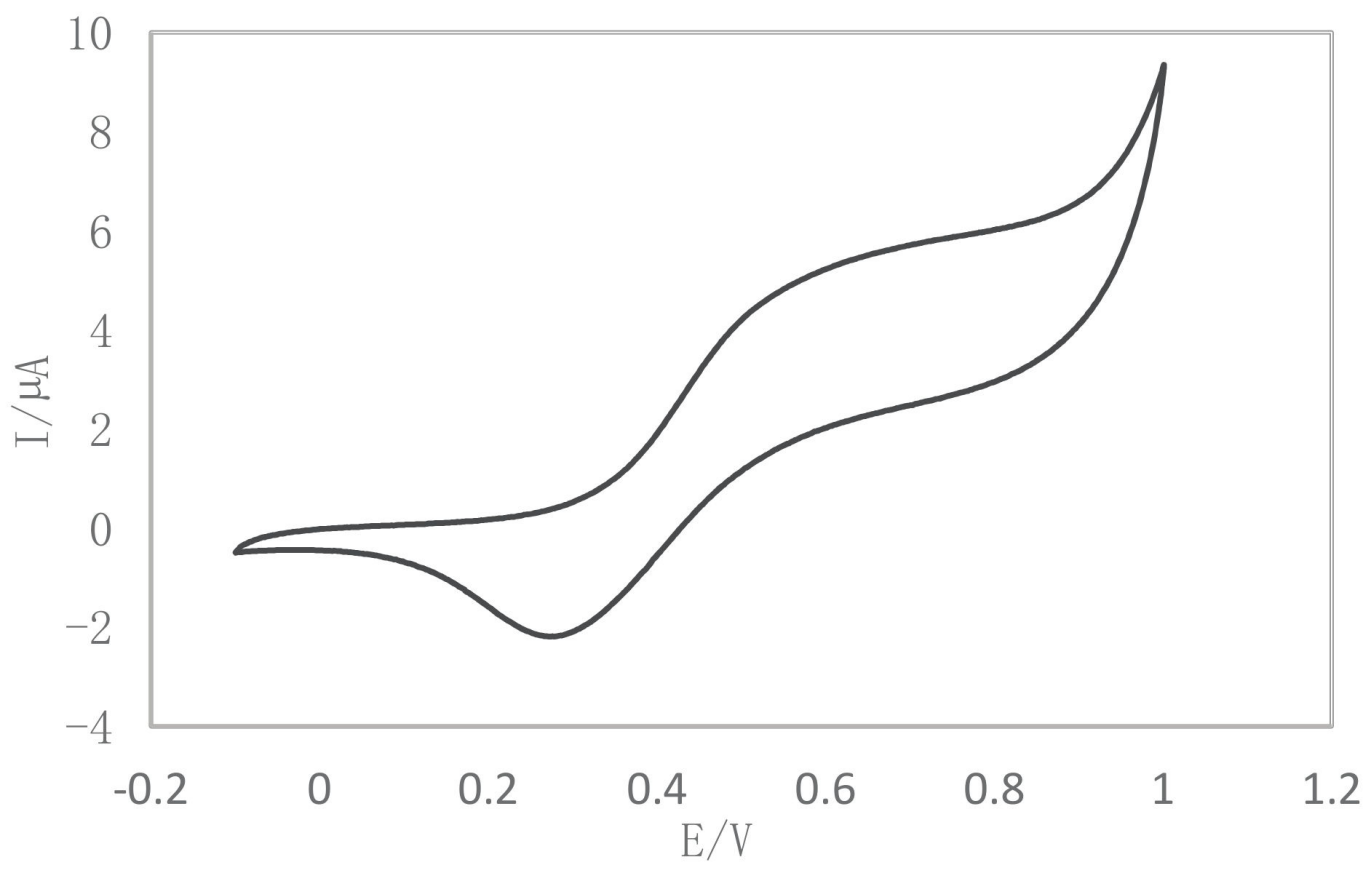
Cyclic voltammetry (CV) of CM extract in pH 7.4 at a scan rate of 30 mV s^−1^.

The reduction behavior of CM extract was evaluated by using cyclic voltammetry (CV), where CM extract exhibited a strong reduction peak and a broad oxidation peak at +0.27 V and +0.2 to +0.8 V vs. Ag/AgCl, respectively (Figure 3). The observed broad oxidation behavior was attributed to the oxidation of a number of antioxidants present in the extract. Low oxidation potential meanwhile demonstrated a stronger antioxidant activity of CM extract [25]. The reduction potential observed for the CM extract at +0.27 V vs. Ag/AgCl correlates with the characteristic peaks of the reduction of terpenoids, which are responsible for the reduction of Au^3+^ ions into Au nanoparticles.

### 2.4. In Vitro Stability Study 

**Figure 4:**
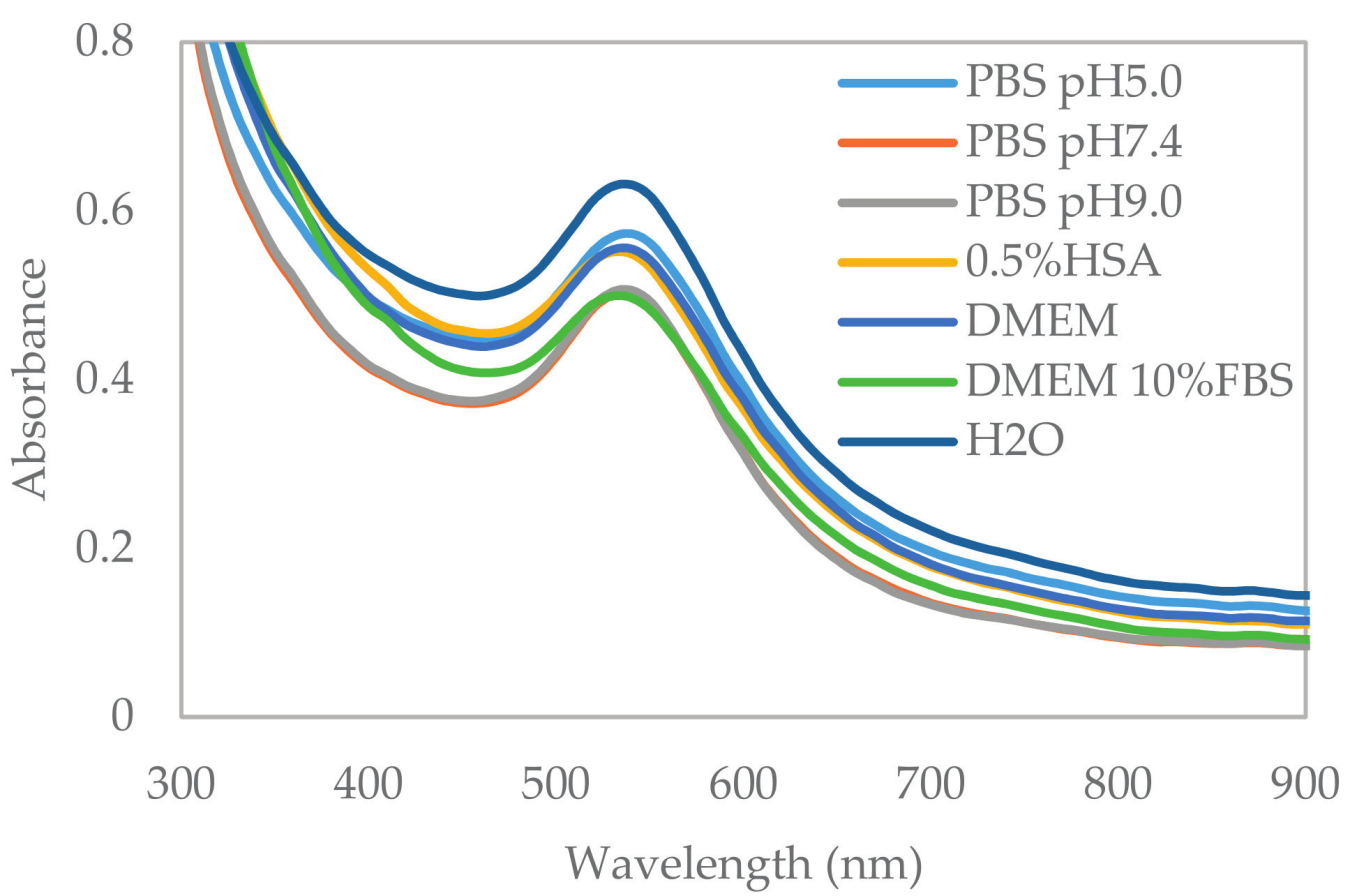
UV-Vis absorption spectra of AuNPs after 24 h of incubation in phosphate buffered saline (PBS) of pH 5.0, 7.4, 9.0, 0.5% human serum albumin (HSA), Dulbecco’s Modified Eagle Medium (DMEM), DMEM with 10% fetal bovine serum (FBS), and ultrapure water.

The stability of AuNPs in buffer and biological media is an essential criteria before using AuNPs for cell-based studies or systemic use. The presence of electrolytes and proteins, for instance, human serum albumin (HSA) in blood stream or fetal bovine serum (FBS) in cell culture media may destabilize the AuNPs [26]. Thus, the stability of AuNPs was investigated by dispersing AuNPs in phosphate buffered saline (PBS) of pH 5.0, 7.4, and 9.0, 0.5% HSA, Dulbecco’s Modified Eagle Medium (DMEM), and DMEM with 10% FBS. Ultrapure water was used as a negative control. The spectra change was monitored by UV-Vis spectroscopy. After 24 h of dispersion, compared with the negative control, no spectral change was observed in the UV-Vis spectra of AuNPs dispersed in the physiological buffer tested (Figure 4).

Zeta potential values of AuNPs in ultrapure water were found to be −38.2 ± 0.6 mV (Table 1). A slight decrease in zeta potential values (−26.4, −29.2, −33.3, and −34.4 mV) were observed for AuNPs dispersed in PBS of pH 5, 7, 9, and 0.5% HSA. Meanwhile, AuNPs in DMEM media and DMEM with 10% FBS demonstrated significant decreases in zeta potential values to −20.9 and −13.5 mV, respectively. 

The hydrodynamic radius of AuNPs in ultrapure water was 78.7 ± 0.5 nm after 24 h. There was discrepancy in size determination using dynamic light scattering (DLS) and TEM analysis, due to the difference in the analytical techniques. DLS measures the hydrodynamic radius of an NP, which includes the coating on the surface of AuNPs, while TEM measures the core size of AuNPs. Therefore, the hydrodynamic radius of a nanoparticle is usually bigger than the core size determined by TEM [27]. The incubation of AuNPs in PBS of pH 5.0, 0.5% HSA, and DMEM media did not cause any significant change in the hydrodynamic size of the AuNPs (Table 1). The incubation of the AuNPs in PBS of pH 7.4 and pH 9.0 decreased the hydrodynamic size of the AuNPs to 55.4 and 68.8 nm, respectively. Meanwhile, the increase in the hydrodynamic size to 114.7 nm was observed for AuNPs incubated in DMEM with 10% FBS [28].

### 2.5. Cytocompatibility Study

Cell viability assay was carried out in order to investigate the in vitro toxicity profile of AuNPs. In this experiment, we used a sulforhodamine B (SRB) assay to determine cell viability. SRB is a negatively charged pink aminoxanthine dye. The SRB assay depends on the ability of SRB to bind to basic amino acid in mild acidic condition and release the dye in a basic condition. SRB is simpler, more sensitive and reproducible, compared to other cytotoxicity assays [29].

**Figure 5:**
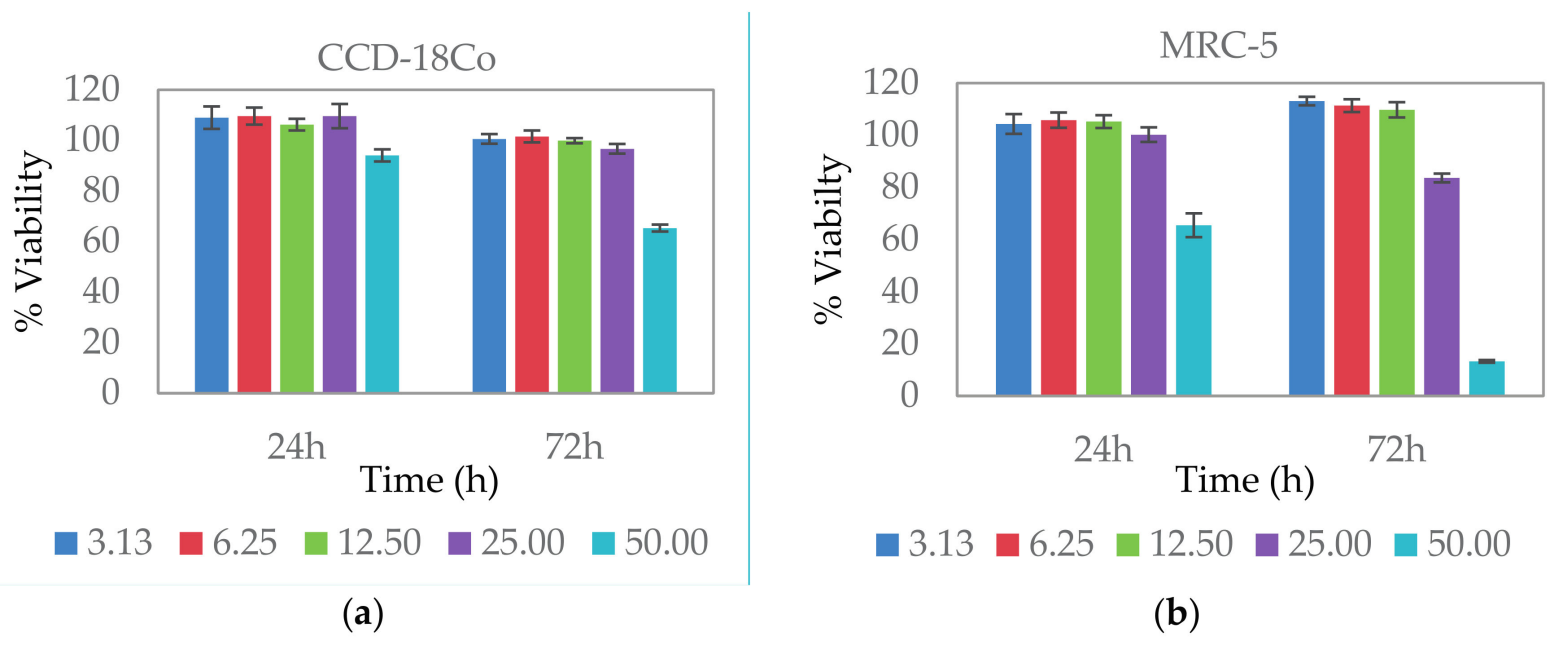

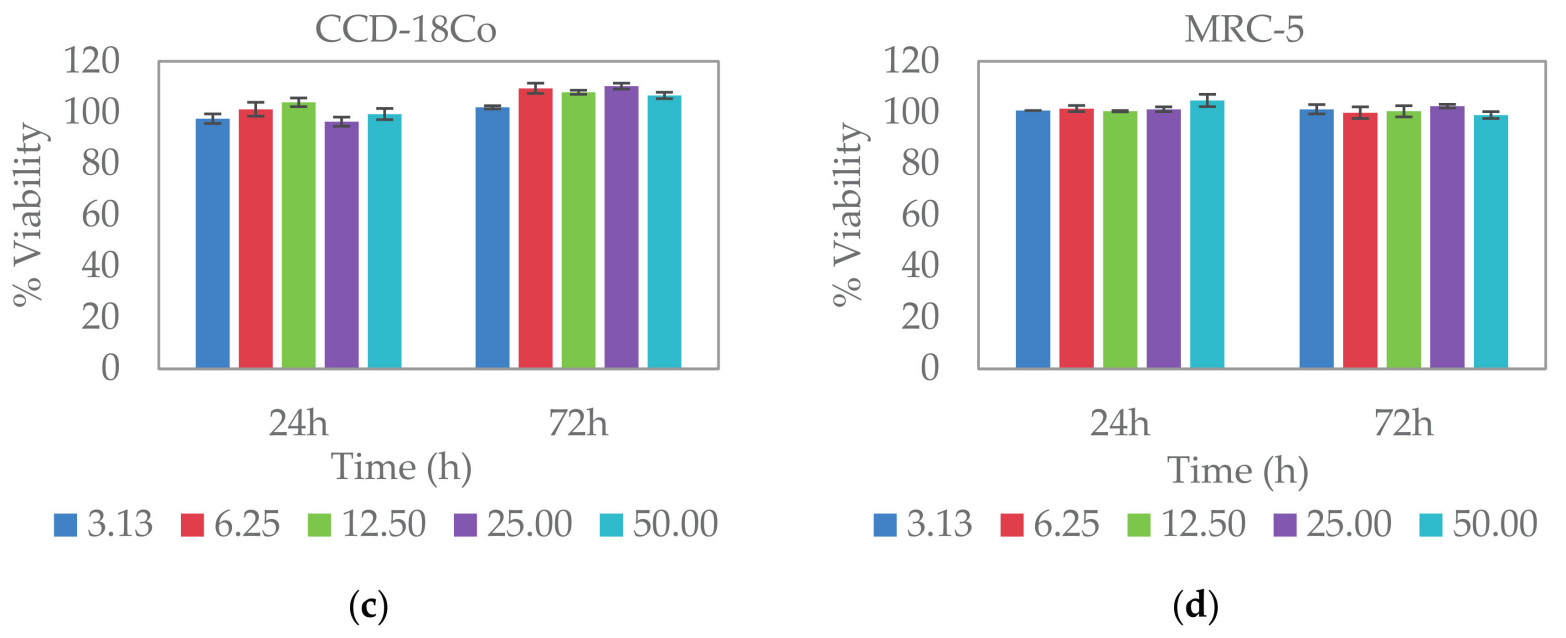
The percentage of viability by the SRB assay for (**a**) human colon fibroblast cell (CCD-18Co) and (**b**) human lung fibroblast cell (MRC-5) after exposure to AuNPs for 24 and 72 h in a dose-dependent manner. The percentage of viability of (**c**) CCD-18Co and (**d**) MRC-5 upon treatment with CM extract for 24 and 72 h. Concentrations of AuNPs and CM extract used in the experiments were 3.13, 6.25, 12.50, 25.00, and 50.00 µg/mL. Results are shown as mean ± S.E. with *n* = 3.

Two normal human fibroblast cell lines were used in this study, which are the human lung fibroblast cell (MRC-5) and human colon fibroblast cell (CCD-18Co). The percentage of viability of CCD-18Co and MRC-5 (Figure 5a,b) at 72 h of exposure to AuNPs at concentrations of 25 µg/mL and lower were maintained above 90% and 80%, respectively. No significant toxic effect to CCD-18Co was observed upon treatment with AuNPs at 50 µg/mL for 24 h, with a percentage of viability of 94.0%. AuNPs only result in a slight inhibition to CCD-18Co after 72 h of treatment with a percentage of viability of 65.3%. AuNPs at high concentration show a more pronounced effect to MRC-5, where 65.4% and 13.3% of cell viability was observed for treatment after 24 and 72 h, respectively. CM extract at concentrations up to 50 µg/mL have not shown any toxic effect to both CCD-18Co and MRC-5 after 24 and 72 h of treatment (Figure 5c,d).

### 2.6. In Vitro Blood Compatibility

**Figure 6:**
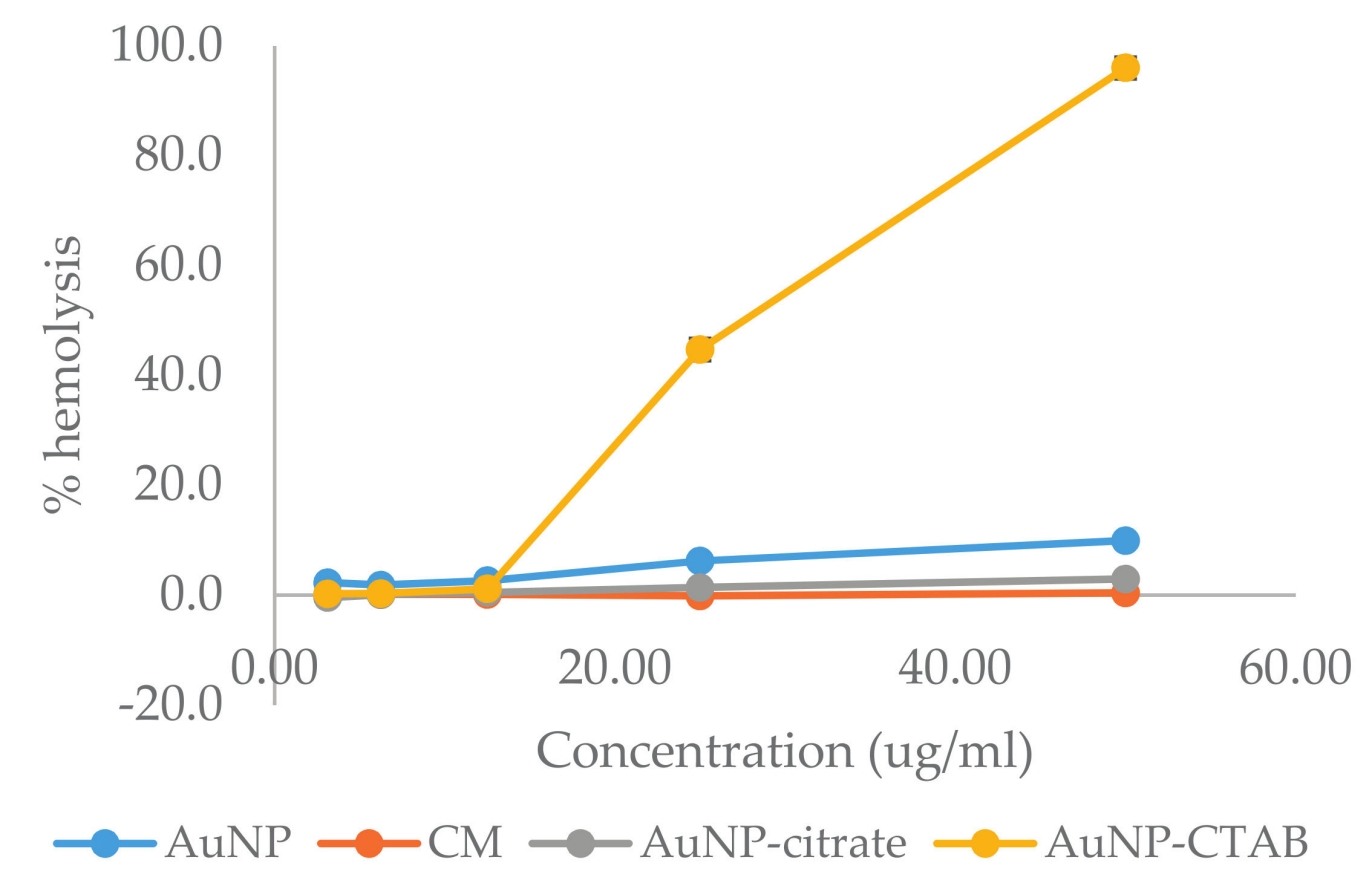
Percentage of hemolysis of red blood cells after exposure to AuNPs at concentrations of 3.13, 6.25, 12.50, 25.00, and 50.00 µg/mL. Each point represents mean ± S.E., with *n* = 3 (if not indicated, the error bar was within the size of the symbol).

Blood compatibility test is an important factor to evaluate toxicity profile of nanoparticles for intravenous use. AuNPs interact with blood cells immediately after administration into biological systems. Thus, it is essential to evaluate hemocompatibility of AuNPs. In this study, hemolysis and the red blood cell (RBC) aggregation test were used to measure the destruction of RBC upon exposure to green-synthesized AuNPs. The hemolysis assay is a colorimetric assay used to estimate the amount of released red-colored hemoglobin, which indicate the extent of the damage of RBC [30]. From Figure 6, the percentage of hemolysis of RBC were found to be 2.7%, 1.8%, 2.6%, 6.2%, and 9.9% upon exposure to AuNPs with concentrations of 3.13, 6.25, 12.50, 25.00, and 50.00 µg/mL, respectively. No hemolysis of RBC was observed upon exposure to CM extract up to concentrations of 50 µg/mL. Citrate-AuNPs results in 2.9% of hemolysis of RBC at a concentration of 50 µg/mL. The exposure of RBC to CTAB-AuNPs of concentrations 25.00 and 50.00 µg/mL results in 44.7% and 96.0% of hemolysis.

**Figure 7:**
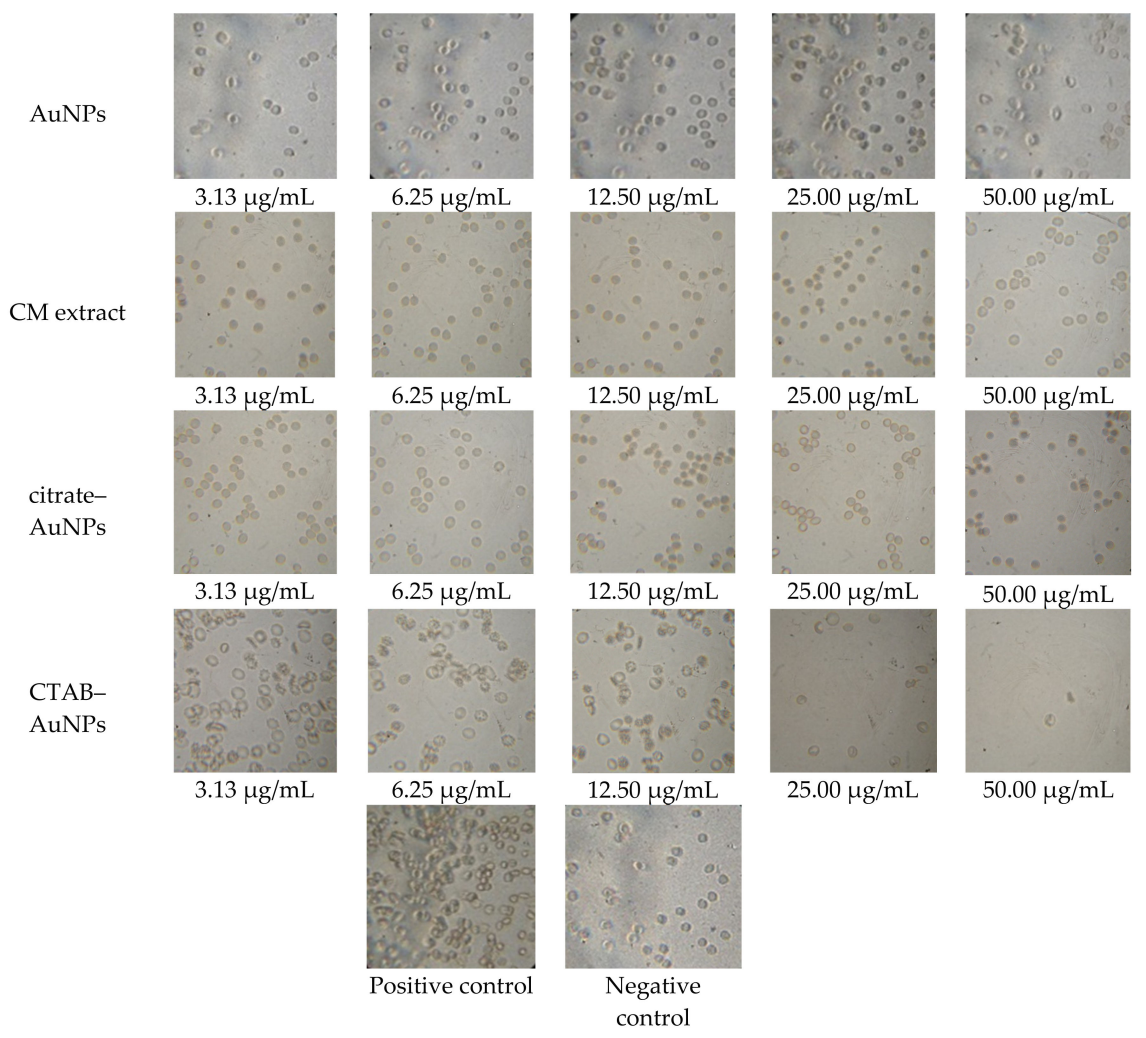
Micrographs of erythrocytes after 2 h of incubation with the positive control, the negative control, the AuNPs, the CM extract, the citrate-AuNPs, and cetyltrimethylammonium bromide (CTAB)-AuNPs at concentrations of 3.13, 6.25, 12.50, 25.00, and 50.00 µg/mL at a magnification of 100×.

Microscopy images in Figure 7 show the aggregation of RBCs upon incubation with the positive control. RBCs treated by AuNPs, CM extract, and citrate-AuNPs maintained healthy smooth biconcave structures, which have a morphology similar to the negative control. No aggregation was observed in the RBCs treated by all concentrations of the AuNPs, CM extract, and citrate-AuNPs tested. Meanwhile, RBC treated with low concentrations (3.13–12.5 µg/mL) of CTAB-AuNPs showed RBC deformation. Lysed RBC was observed upon treatment with CTAB-AuNPs with a concentration of 25 µg/mL. Only cell debris can be seen for RBC treated with 50 µg/mL of CTAB-AuNPs since almost all of the RBC underwent hemolysis.

## 3. Discussion

The applications of AuNPs have shown promising advancements in the field of medicine. However, very few AuNPs have gone through clinical trials and have proven to be beneficial for human applications, due to the lack of knowledge in the physical characteristics of AuNPs in the physiological system. This study therefore involves in vitro stability, cytocompatibility, and hemocompatibility investigations as a prerequisite evaluation on AuNPs for in vivo applications. 

A simple, one-step method was demonstrated for the green synthesis of AuNPs using CM extract. This method is fast, cheap, and energy-efficient, where no heating and stabilizing agent was required for the formation of AuNPs. A bottom-up approach, where Au^3+^ ions from chloroauric acid (HAuCl_4_·3H_2_O) act as the building blocks for the formation of AuNPs, was adopted [13]. The antioxidants in CM extract serve as a reducing agent in the synthesis of AuNPs [31]. From FTIR analysis, carbonyl groups in the CM extract were shown to be involved in the reduction of Au^3+^ ions. CM extract is composed of terpenoids, for instance, labda-8(17), 12-diene-15,16-dial, 15,16-bisnor-labda-8(17),11-diene-13-on, calcatarin, and Zerumin A [18,19]. Labda-8(17), 12-diene-15,16-dial, calcatarin, and Zerumin A have aldehyde groups, while 15,16-bisnor-labda-8(17),11-diene-13-on has a keto group. These compounds are capable of reducing the Au^3+^ to Au^0^. This result is consistent with a previous study that showed predominant involvement of carbonyl group in the reduction of Au^3+^ ions [32]. 

**Scheme 1:**
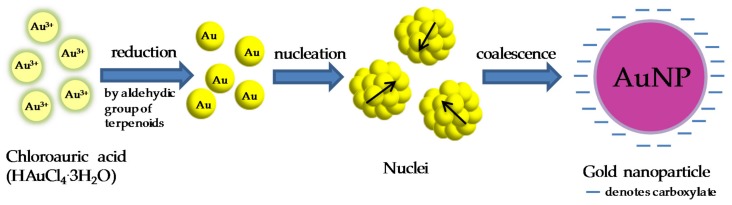
The proposed mechanism for the formation of AuNPs synthesized using CM extract.

The reduction capability of CMEW extract in reducing the Au^3+^ ions into AuNPs was further supported by cyclic voltammetry analysis. As clearly depicted in Figure 3, the reduction potential was observed for CM extract at +0.27 V vs. Ag/AgCl, which is well correlated with the characteristic reduction potential of terpenoids. The obtained cyclic voltammogram has demonstrated the reduction capability of terpenoids in CM extract for the reduction of Au^3+^ ions into AuNPs. We propose a mechanism for the formation of AuNPs as shown in Scheme 1. The reduction of Au^3+^ to Au leads to the formation of nuclei. The nuclei coalesce and contribute to the growth of AuNPs [33]. Terpenoids with carboxylic groups, such as Longpene A, Coronadiene, and Zerumin A can bind to AuNP surfaces and act as a stabilizing agent [34].

The evaluation on the stability of AuNPs in biological media is important to predict the fate of AuNPs after systemic administration. Bloodstream or physiological media contain serum proteins, electrolytes, nutrients, and metabolites that contribute to a high ionic strength. The aggregation of chemically synthesized AuNPs may occur when they were subject to the blood stream, limiting the in vivo human applications of AuNPs [6]. Chemically synthesized AuNPs, such as citrate-capped AuNPs and CTAB-coated AuNPs, are unstable and form aggregates in physiological media [7,35]. Wang et al. reported a rapid drop in absorption intensities of citrate-capped and CTAB-coated AuNPs in the first 15 min of incubation in PBS of pH 4, 7, 8, and 10, which indicate the instability of the AuNPs in PBS. Another study has shown that diluting citrate-capped AuNPs in PBS results in the formation of agglomerates 2–3 times larger in size [36]. In our experiment, the dispersion of AuNPs in PBS, 0.5% HSA, DMEM, and DMEM with 10% FBS did not alter the UV-Vis spectra, suggesting that the green-synthesized AuNPs were stable in all conditions tested. There were also no significant changes in the size of AuNPs upon their dispersion in PBS, the HSA solution, and DMEM, suggesting that AuNPs synthesized from CM extract are stable. The 46% increase in size of AuNPs after 24 h of dispersion in DMEM with 10% of FBS due to the formation of protein corona on the surface of AuNPs contributed by FBS. This is a common phenomenon where instantaneous absorption of protein occurred once the nanoparticles were exposed to media containing protein [5].

The zeta potential values decreased by 12.8 to 30.9% upon incubation with PBS, which might be due to the high ionic strength of PBS disrupting the surface charge of AuNPs. The zeta potential values become less negative as the pH of PBS decreased, which is due to the higher concentration of H^+^ ions in acidic PBS, which reduced the negative surface charge of AuNPs [37]. The significant reduction in zeta potential values of AuNPs in DMEM media is caused by a high ionic environment in the media. DMEM media contain a high percentage of inorganic salts, amino acids, and vitamins [5]. The alteration in zeta potential, however, did not affect the stability of AuNPs in all physiological media, as demonstrated by UV-Vis spectroscopy and hydrodynamic size results. All these data support the fact that AuNPs synthesized by CM extract were stable in all physiological conditions tested. 

AuNPs synthesized by CM extract were biocompatible. Cytocompatibility results suggested that AuNPs synthesized from CM extract did not have toxic effects on the two normal cells tested at the concentration of 25 µg/mL. Therefore, AuNPs are non-toxic for biomedical applications. The cytocompatibility of AuNPs was attributed to the biocompatibility of CM extract used as a stabilizing agent. In the preparation of AuNPs via chemical synthesis, the residual contamination from the synthesis or the desorption of the stabilizing agent from AuNPs may contribute to the toxicity of AuNPs. A common capping agent for gold nanorods, CTAB, has a high cytotoxicity against cells at nanomolar concentrations. This resulted in a significant cytotoxicity of CTAB-capped gold nanorods. As such, overcoating the CTAB-capped gold nanorods with polymers was required to reduce the toxicity of CTAB-capped gold nanorods [38]. Herein, we demonstrated that the use of phytochemicals as capping agents might be a better option for the synthesis of biocompatible AuNPs. 

The investigation of toxicity towards RBC is an important parameter to evaluate the safety and circulation characteristics of AuNPs. This study has shown that AuNPs synthesized from CM extract are hemocompatible. No more than 10% of hemolysis was observed for all tested concentrations up to 50 µg/mL, which is below the safe hemolytic ratio for biomaterials according to ISO/TR7406 [39]. The aggregation of RBC was also not observed for all concentrations of AuNPs tested. The hemocompatible characteristic of AuNPs is attributed to its negative zeta potential and hemocompatibility of the CM extract. Meanwhile, we have shown that CTAB-AuNPs are not blood compatible, where 44.7% and 96.0% of hemolysis was observed upon treatment with 25 and 50 µg/mL CTAB-AuNPs. This may be due to desorption of CTAB from CTAB-AuNPs. Free CTAB molecules can cause lysis to RBC at low concentrations [40]. Thus, CTAB-AuNPs is not recommended for biomedical applications.

## 4. Materials and Methods 

### 4.1. Materials

Gold (III) chloride trihydrate (HAuCl_4_·3H_2_O), citrate-stabilized gold nanoparticles (20 nm), Eagle’s Minimum Essential Medium (EMEM), fetal bovine serum (FBS), sodium pyruvate, amphotericin B, penicillin/streptomycin, human serum albumin (HSA), and sulforhodamine B (SRB) dye were purchased from Sigma-Aldrich (Saint Louis, MO, USA). The human normal colon fibroblast cell line (CCD-18Co) and the human lung fibroblast cell line (MRC-5) were purchased from American Type Culture Collection (ATCC, Manassas, VA, USA). Dulbecco’s Modified Eagle Medium (DMEM) without phenol red was purchased from Nacalai (Kyoto, Japan). Fresh human blood was collected in EDTA tubes. The blood was centrifuged at 4 °C, 1000× *g* for 10 min to obtain red blood cells (RBC). *C. mangga* rhizomes were obtained from Yogjakarta, Indonesia. CM extract was obtained through the extraction of *C. mangga* rhizomes by 50% aqueous ethanol and dried under vacuum evaporator.

### 4.2. Synthesis and Purification of AuNPs

A stock solution of 10.0 mg/mL of the CM extract in 50% aqueous ethanol was prepared for the synthesis of AuNPs. The AuNPs were prepared as previously described [41]. Briefly, 2 mL of CM extract was added to 5 mL of 1 mM aqueous chloroauric acid (HAuCl_4_·3H_2_O) and the mixture was allowed to incubate at room temperature. The formation of AuNPs was indicated by the change of color of the suspension from yellow to red-violet. The AuNPs produced were then purified by repeated centrifugation at 14,000 rpm for 30 min at 4 °C, after which AuNP pellets were suspended in ultrapure water. The microwave plasma-atomic emission spectroscopy (MP-AES) (Agilent Technologies, Santa Cara, CA, USA) technique was utilized to determine the final concentration of AuNPs in the suspension.

### 4.3. Characterization of AuNPs 

UV-Vis absorption spectra of AuNPs were measured by Synergy H1 hybrid reader (BioTek, VT, USA) at a resolution of 1.0 nm between 300 and 800 nm. Transmission electron microscopy (TEM) measurements were carried out using JEM-7600F high resolution-TEM (JEOL, Tokyo, Japan) with an accelerating voltage of 120 kV. TEM samples were prepared by dropping as-synthesized AuNPs on carbon-coated copper grid and allowed to dry in the oven overnight. The size distribution was determined by measuring the diameter of about 400 particles using Image J software (National Institute of Health, Bethesda, MD, USA). Fourier transform infrared (FTIR) spectroscopy was performed to determine the possible biomolecules involved in the synthesis of AuNPs. FTIR spectra of CM extract and CM after the reduction of Au^3+^ were measured using a Spectrum 400 (PerkinElmer, Boston, MA, USA) in the spectral range of 4000–450 cm^−1^ with a resolution of 1 cm^−1^. Cyclic voltammetry (CV) measurement was carried out with the aid of a three-electrode configuration cell comprising an indium tin oxide (ITO) working electrode of (1 × 2) cm dimension, an Ag/AgCl reference electrode, and a Pt wire counter electrode. The three-electrode configuration cell was placed in 0.2 wt% CM extract with a 0.2 M phosphate buffer solution (pH = 6.6). CV measurements were performed using a multi-channel Autolab (PGSTAT30) potentiostat workstation (Metrohm Autolab, Utrecht, Netherlands) under a potential range of −0.1 to 1.0 V vs. Ag/AgCl and at a scan rate of 30 mV/s. 

### 4.4. In Vitro Stability Study 

In vitro stability study was performed as described by Basha et al. with modification [16]. Briefly, 0.5 mL of AuNP suspension were added to 0.5 mL of 0.5% HSA, phosphate buffer saline at pH 5.0, 7.4, and 9.0, DMEM media, and DMEM with 10% FBS, respectively. A total of 0.5 mL of ultrapure water was used as control. The change in the absorbance spectra of AuNPs after 24 h was monitored using UV-Vis spectroscopy. Hydrodynamic size and zeta potential of AuNPs was measured using a Zetasizer Nano ZS (Malvern Instruments, Malvern, UK) operated at 25 °C.

### 4.5. Cytocompatibility Study

CCD-18Co and MRC-5 were maintained in the EMEM medium supplemented with 10% FBS, 1% sodium pyruvate, amphotericin B, and penicillin/streptomycin, respectively, incubated at 37 °C in a 5% CO_2_ incubator. When the cells reached 70–80% confluency, the cells were plated in 96-well plate with 8000 cells/well for 24 h. The cells were then incubated with AuNPs and CM extract at concentrations of 3.13, 6.25, 12.50, 25.00, and 50.00 µg/mL for 24 and 72 h. Cell viability was determined by the SRB assay as described by Skehan et al. [42] The absorbance values of SRB were obtained using the Synergy H1 hybrid reader (BioTek, Winooski, VT, USA) at a wavelength of 492 nm.

### 4.6. Hemocompatibility Test

Hemolysis assay was conducted as described by Evans et al. [43] with modification. One hundred microliters of 2% RBC were mixed with 100 µL of AuNPs, CM extract, citrate-AuNPs, and CTAB-AuNPs of different concentrations in PBS. PBS and 1% *v*/*v* Triton-X were used as negative and positive controls, respectively. Absorbance of hemoglobin released was measured at 550 nm after 5 h of incubation at 37 °C. The percentage of hemolysis was calculated using absorbance of positive control as 100% hemolysis. The aggregation test was conducted according to Singhal and Ray with modification [44]. One hundred microliters of 2% RBC suspension were mixed with 100 µL of AuNPs, CM extract, citrate-AuNPs and CTAB-AuNPs of different concentrations in PBS. RBC at 2% concentration, suspended in anti-coagulant (sodium citrate), was used as the negative control, while the positive control was prepared as a solution with no sodium citrate. Photomicrographs of RBC were obtained at 100× magnification after incubation at 37 °C for 2 h.

## 5. Conclusions

We demonstrated a successful green synthesis of AuNPs from precursor Au^3+^ ions mediated by CM extract. This one-step synthesis facilitates easier and practically economic routes for industrial scale-up production with reduced environmental risks. In vitro stability studies show that the AuNPs were stable in PBS of pH 5, 7, and 9, HSA solution, and DMEM media. Slight alterations in size and zeta potential value were observed in AuNPs dispersed in DMEM with 10% FBS. AuNPs synthesized by CM extract also demonstrated good cytocompatibility and blood compatibility. Generally, the positive outcomes of the work warrant the need for further investigations using animal models for a clinical translation of AuNPs.

## Figures and Tables

**Table 1:** Zeta potential values and hydrodynamic sizes of gold nanoparticles after 24 h of incubation in PBS of pH 5.0, 7.4, and 9.0, 0.5% human serum albumin (HAS), Dulbecco’s Modified Eagle Medium (DMEM) media, and DMEM media with 10% fetal bovine serum (FBS).

Physiological Media	Zeta Potential (mV)	Size (nm)
PBS pH 5.0	−26.4 ± 1.0 *	82.9 ± 1.0
PBS pH 7.4	−29.2 ± 0.8 *	55.4 ± 1.5 *
PBS pH 9.0	−33.3 ± 0.6 *	68.8 ± 2.0 *
0.5% HSA	−34.4 ± 1.0 *	77.3 ± 1.3
DMEM	−20.9 ± 0.4 *	75.8 ± 1.8
DMEM with 10% FBS	−13.5 ± 0.1 *	114.7 ± 1.4 *
Control	−38.2 ± 0.6	78.7 ± 0.5

Results presented as mean ± S.E, with *n* = 3. * significant difference compared to control at *p* < 0.05, obtained by one-way ANOVA.
